# The Year of the Rat: The Rat Genome Database at 20: a multi-species knowledgebase and analysis platform

**DOI:** 10.1093/nar/gkz1041

**Published:** 2019-11-12

**Authors:** Jennifer R Smith, G Thomas Hayman, Shur-Jen Wang, Stanley J F Laulederkind, Matthew J Hoffman, Mary L Kaldunski, Monika Tutaj, Jyothi Thota, Harika S Nalabolu, Santoshi L R Ellanki, Marek A Tutaj, Jeffrey L De Pons, Anne E Kwitek, Melinda R Dwinell, Mary E Shimoyama

**Affiliations:** 1 Rat Genome Database, Department of Biomedical Engineering, Medical College of Wisconsin, Milwaukee, WI 53226, USA; 2 Genomic Sciences and Precision Medicine Center and Department of Physiology, Medical College of Wisconsin, Milwaukee, WI 53226, USA

## Abstract

Formed in late 1999, the Rat Genome Database (RGD, https://rgd.mcw.edu) will be 20 in 2020, the Year of the Rat. Because the laboratory rat, *Rattus norvegicus*, has been used as a model for complex human diseases such as cardiovascular disease, diabetes, cancer, neurological disorders and arthritis, among others, for >150 years, RGD has always been disease-focused and committed to providing data and tools for researchers doing comparative genomics and translational studies. At its inception, before the sequencing of the rat genome, RGD started with only a few data types localized on genetic and radiation hybrid (RH) maps and offered only a few tools for querying and consolidating that data. Since that time, RGD has expanded to include a wealth of structured and standardized genetic, genomic, phenotypic, and disease-related data for eight species, and a suite of innovative tools for querying, analyzing and visualizing this data. This article provides an overview of recent substantial additions and improvements to RGD’s data and tools that can assist researchers in finding and utilizing the data they need, whether their goal is to develop new precision models of disease or to more fully explore emerging details within a system or across multiple systems.

## INTRODUCTION

**Figure 1.**
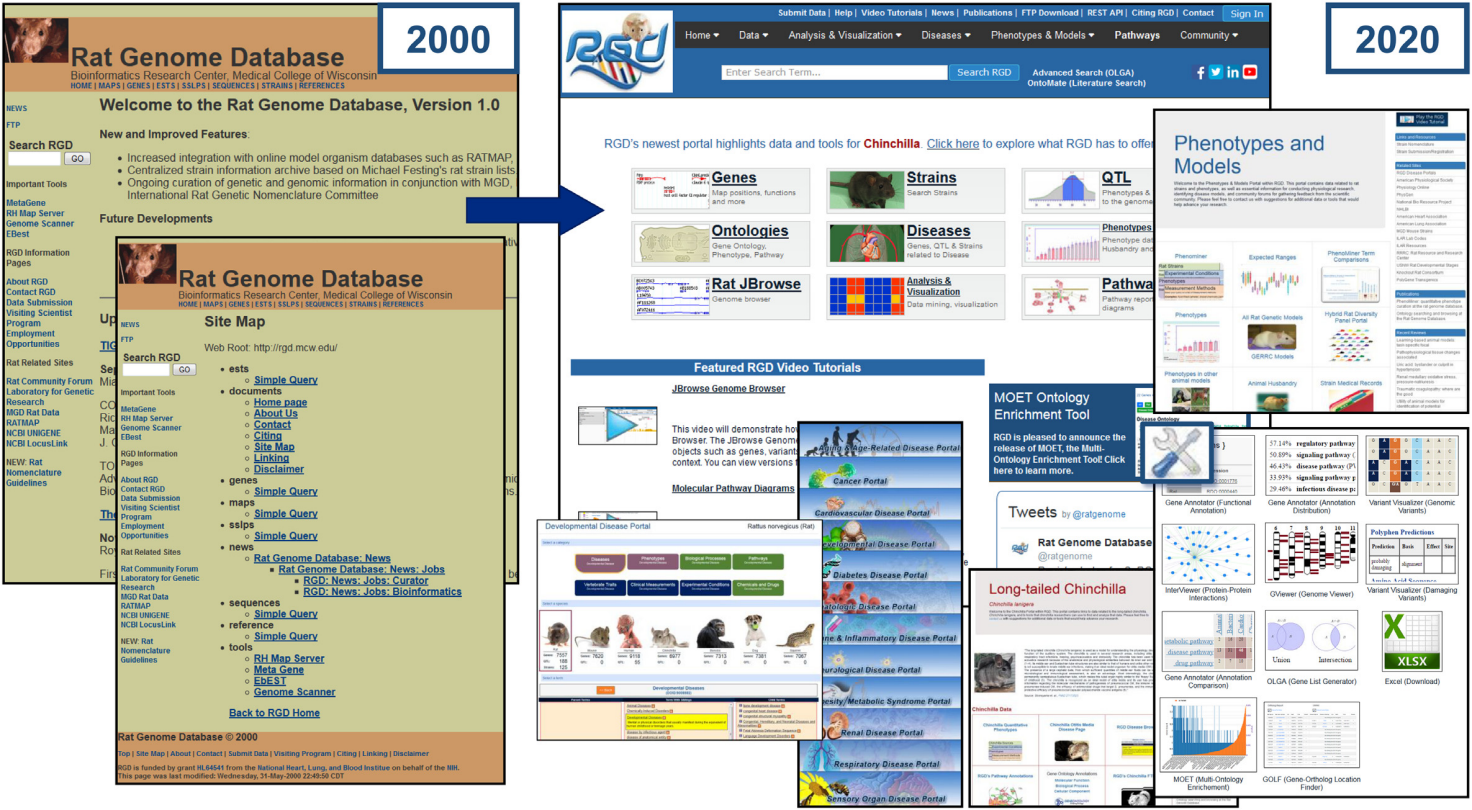
The Rat Genome Database at 20. RGD has evolved over twenty years from a simple data repository offering a few data types and a small number of tools, to a multispecies knowledgebase offering numerous data types, utilizing 15 ontologies, housing over 130 000 references used in house for creating manual annotations across data types and species, and integrating a wealth of data imported by over 80 automated pipelines. In addition, RGD has developed a suite of innovative tools for searching, visualization and analysis.

The Rat Genome Database (RGD, https://rgd.mcw.edu) was created in late 1999, and rapidly became the premier online location for genetic, genomic, phenotypic and disease-related data for the laboratory rat, *Rattus norvegicus*, as well as a source of comparative data for rat, mouse and human. In contrast to the current wealth of data and tools, the ‘bare bones’ first iteration (Figure 1, Table 1) focused on just a few data types—most notably a handful of known genes, plus EST and SSLP markers—localized only on genetic and RH maps, and offered a minimal number of tools for searching and analyzing that data.

**Table 1.** Comparison of the number of data records in RGD from 2000 to 2019 by data type and species

SPECIES	Rat		Human		Mouse		Chinchilla	Bonobo	Dog	Squirrel	Pig
Year	2000	2019	2000	2019	2000	2019	2019	2019	2019	2019	2019
GENES	1987	45 816	14	40 984	857	53 724	29 971	33 712	36 850	26 325	30 414
MARKERS (SSLPS + ESTS)	19 562	50 130		3 20 143		55 165					
STRAINS	76	3740									
QTLS		2378		1911		6335					
PROTEINS		36 262		1 82 299		88 294	99	43 648	29 696	25 485	3 33 908
MAPS/ASSEMBLIES	5	12	1	19		11	1	2	2	1	3
CELL_LINES		41									
PROMOTERS		12 720		63 992		57 546			7 545		
TRANSCRIPTS		1 64 769		2 11 136		1 78 349	75 934	62 481	98 852	50 117	75 517
VARIANTS		>600 000 000		6 00 981							
REFERENCES (species is not assigned for references)	12	1 31 295									

In 2000, the major data type in RGD was rat markers, comprised of both expressed sequence tags (ESTs) and simple sequence length polymorphisms (SSLPs). The maps in 2000 included only genetic and RH maps and the cytogenetic map. In 2019, RGD stores and presents data across eight species, with genes, transcripts and proteins for all species, and variants for rat and human comprising the largest datasets. Note that RGD does not currently track rat variants across assemblies or between strains.

Even before the first public release of the rat reference genome in 2002 (1), RGD was focused on mapping disease-related regions and on comparative genomics between rat, human and mouse to support the use of rat as a model for human disease. The first version of the Virtual Comparative Map (VCMap) tool was mentioned in the first of RGD’s Nucleic Acids Research Database issue articles in 2002 (2). In that version, VCMap's comparisons were based on finding homologous ESTs and cDNAs between rat, mouse and human and using RH maps and UniGene clusters to build the comparative maps. By 2005, RGD had added quantitative trait loci (QTLs) to VCMap (3). In addition, by 2005 RGD had implemented curation of biological data using multiple ontologies to give researchers a more complete view of the functionality of each gene, QTL or strain (4). The four original ontologies that RGD used were the Gene Ontology (GO), the Mammalian Phenotype ontology (MP), a Disease Ontology (RDO) based on MeSH, and the Pathway Ontology (PW) under development at RGD.

RGD’s first automated ingest pipeline to import gene records from NCBI was developed in 2006. Previous to that, the gene records were imported using a ‘semi-automated’ approach which required several weeks to run and substantial work by curators to review each record before the dataset could be loaded into the database. Because of the time and manual effort required, gene records were only updated 2–3 times a year, causing inconsistencies between the records at RGD and those at NCBI. In contrast, the new pipeline was able to complete the load process in two days, could be run on a weekly basis and required no curator input beyond a review of the conflicts. Such conflicts were reviewed and either corrections were made to the data or the pipeline algorithm was adjusted so that over time the number of conflicts was substantially reduced. In the years since then, RGD has implemented and currently runs >80 pipelines. These pipelines regularly import data from external databases, export data to RGD’s FTP site for uptake by other databases and research groups and perform QC or analysis on data in the RGD database.

RGD’s first two disease portals, the Neurological Disease portal and the Cardiovascular Disease portal, were released in 2006 (5). In 2008, the Phenotypes and Models portal was created to facilitate researchers' access to physiological data (6). The first version of this portal included quantitative phenotype data from the PhysGen Program for Genomic Applications project and a section of information about rat strains and models, including information about established model strains for several disease categories and a list of strain availabilities. RGD’s interactive pathway diagrams were first announced in 2009 (6) with the pathway portal release not far behind (7). In 2012–2013, RGD began the PhenoMiner project to standardize quantitative phenotype data using a suite of ontologies developed at RGD (8,9). The large corpus of high-throughput phenotype measurement data from PhysGen constituted the initial dataset loaded into the tool.

RGD had begun dealing with genome-level variant data by 2009 (6) with the import of variants from the STAR Consortium and the development of the SNPlotyper tool to query this data. In 2013, Atanur *et al.* (10) published the whole genome sequencing of 27 rat strains. The almost 13 million variants from this sequencing project were submitted to RGD prior to publication. This dataset was too large to be able to use the SNPlotyper tool for querying and analysis, prompting the development of the Variant Visualizer. Since its first release, the Variant Visualizer has been expanded to include rat variants for the three major assemblies (RGSC3.4, Rnor5.0 and Rnor6.0), encompassing data for 72 unique strains or substrains, as well as human variants from the ClinVar database at NCBI for the GRCh37 and GRCh38 assembly builds.

This retrospective highlights RGD’s early commitment to integrating data and providing tools which support and facilitate disease research, comparative and translational studies, and cross-species analyses. In the past 20 years, this focus has not shifted to other subjects. RGD’s primary goal remains to not only maintain but to expand the data and tools provided to support disease research. In recent years, substantial attention has been paid to the selection of appropriate model systems to study human disease. For some specific diseases, review articles provide information about the pros and cons of various models [e.g., (11–19)]. But such resources are not universally available and are not necessarily updated as new models emerge. On the other hand, model organism databases (MODs) such as RGD, store and present structured, standardized data that researchers can use to assess applicable models, and are updated on an ongoing basis to keep the data as consistent as possible with current information.

Since the previous update in 2015 (20), RGD has added data for five additional mammalian species (chinchilla, bonobo, 13-lined ground squirrel, dog and pig). Genome information pages provide consolidated information about the genome and the state of the assembly for each of RGD’s eight species. Additional automated pipelines now bring in protein-protein interaction data from the IMEx Consortium (21), as well as an improved ortholog set and improved gene descriptions from the Alliance of Genome Resources, of which RGD is a founding member (see below). REST APIs and species-specific FTP directories facilitate manual and programmatic access to RGD data at scale. In addition, RGD’s toolbox has been expanded to include six new tools for finding and analyzing gene sets, visualizing protein-protein interactions, comparing quantitative phenotype data across rat strains, and searching for publications using ontology tags. The addition of links that directly submit the results obtained with one tool as input for another tool facilitates navigation between tools, simplifying researchers' analysis workflows and helping users assess the most efficacious next step in their analysis. RGD’s disease portals have been updated, increasing both their flexibility and their functionality. RGD’s genome browser has been upgraded from GBrowse (22) to the faster and more agile JBrowse browser (23), with implementations provided for all of RGD’s species. This article highlights these recent improvements as well as pointing out some of the ways in which RGD has expanded its collaborations with other major model organism databases, data warehouses and knowledgebases. These improvements—the ongoing expansion of available data, additions and improvements to the RGD tool suite, and integration into the larger research and bioinformatics communities—enable RGD to provide data and tools that help researchers to most effectively and efficiently answer their research questions and to determine the best precision model for their human disease of interest.

## NEW DATA

### Additional species

The rat has been studied as a model for human physiology and disease for over 160 years. Historically, the rat's physiological similarity to human and the ability of researchers to produce strains that develop abnormal conditions which more or less mimic human diseases such as cardiovascular diseases, diabetes, kidney disease, arthritis, neurodegenerative diseases, etc., combined with its tractable size and behavior, and relatively short reproductive cycle made it the model of choice for many human conditions. It is not, however, the best model in every case. As concern increases over the perceived (or proven) inappropriateness of rodent models for certain diseases, researchers need to be able to determine and utilize more precise models for their disease of interest, that is, models that more closely mimic the human phenotypic profile for a disease.

In addition, researchers who use rat for their studies often also utilize other species such as mouse to best answer their research questions. To facilitate such cross-species (e.g. rat/mouse) and translational (model organism/human) analyses, RGD has always provided data for mouse and human, in addition to rat. This commitment to providing data across species prompted the development of automated pipelines to import data such as Gene Ontology (GO) annotations for mouse and human genes, manual disease annotations from other databases such as OMIM, and the extensive set of manual phenotype annotations for mouse genes from the Mouse Genome Informatics (MGI) (24) database. Such pipelines are able to leverage RGD’s store of external database identifiers and use of ontologies to match the incoming data to existing records and integrate the two. Standardization of these pipelines allows for efficient development of new pipelines to bring in novel data. Because of this, RGD has been able to expand its repertoire of species to include chinchilla, bonobo, 13-lined ground squirrel, dog and pig, in addition to rat, human and mouse. In each case, the species is a well-studied model for one or more human diseases.

Because the inner and middle ear and Eustachian tube structures of the long-tailed chinchilla (*Chinchilla lanigera*) closely resemble those of human, chinchilla is commonly used for the study of hearing, psychoacoustics, ototoxicity and upper respiratory tract infections. In addition, due to its relative resistance to innate infections of the middle ear compared to other rodent models, it is the model of choice for the study of bacterial and viral infections of the middle ear (otitis media) (25–29).

The cone-dominant retina of the 13-lined ground squirrel (*Ictidomys tridecemlineatus*) and the squirrel's diurnal habits make squirrel a better model for studies of visual processing and retinal function and dysfunction than either mouse or rat (30). In addition, the 13-lined ground squirrel is an obligate hibernator. Its ability to survive, and even thrive, through repeated cycles of long periods of hypometabolic torpor interspersed with brief periods of euthermic arousal during hibernation makes it an excellent model for studies of hypoxia/reperfusion, metabolism, and longevity (31).

Like many other primates, including human, the bonobo, also known as the pygmy chimpanzee (*Pan paniscus*), can develop a number of cardiovascular diseases including hypertension, cardiomyopathy with fibrosis, congestive heart failure, aortic dissection, stroke and arrhythmogenic right ventricular cardiomyopathy (ARVC) with associated sudden death. In fact, the incidence of these diseases is strikingly high among captive ape populations making bonobo both a good model for human cardiovascular disease and an urgent subject of research on the causes of and treatments for its own cardiovascular diseases (32–34).

Dogs (*Canis lupus familiaris*) are studied both as models for human disease and to improve their health, happiness and longevity as companion animals. Centuries of breeding, and in many cases inbreeding, have resulted in extensive variation in genetics, and behavioral and morphological phenotypes. Certain breeds of dogs are susceptible to inherited and/or environmentally-induced conditions that also affect humans including cancers, cardiovascular disease, rheumatoid arthritis and other autoimmune diseases, neurological disorders, deafness and blindness (35–40).

Although most commonly thought of as agricultural animals, pigs (*Sus scrofa*) are also used in biomedical research in studies of physiology and disease. Anatomical, metabolic and physiological similarities between pigs and humans make pig a good model for human cardiovascular disease, obesity, diabetes, kidney disease, respiratory disease and organ transplantation (41–46). In addition, the pig's omnivorous diet and digestive physiology (e.g. colonic fermentation) make it a suitable host for studies of the human microbiome (47,48).

For each of these species, RGD has imported gene records from NCBI (49) and, where applicable, Ensembl (50). For species with protein information in UniProtKB (51), this protein data has been imported and assigned to the corresponding genes in RGD. Functional data in the form of disease, gene ontology, and pathway ontology annotations have been informatically ‘inferred’ to these genes from their orthologs in human, mouse and rat.

In addition to annotations which are inferred from other species, in several cases RGD has been able to assign species-specific annotations to genes in these ‘new’ species. For instance, because of the small volume of literature with functional information for chinchilla genes, RGD curators were able to review all of the available literature and made manual annotations for chinchilla genes. As most research results for chinchilla are phenotype-based rather than genome- or gene-based, this effort resulted in 39 experimentally-derived (that is, not based on orthology to genes in another species) disease annotations and 120 experimental GO annotations. On the other hand, the research emphasis on characterization of phenotypes in the chinchilla such as susceptibility to infection and tissue-specific responses to inflammation prompted the addition of quantitative phenotype data for chinchilla to RGD’s PhenoMiner tool (8). Several proof-of-concept datasets have been added, including measurements of bacterial counts and tympanic cavity epithelium thickness during middle ear infections and urinalysis data from healthy chinchillas. Being raised in small colonies, chinchillas are relatively outbred but not as genetically diverse as would be expected of a wild population with many members, so a chinchilla ‘source’ designation has replaced the rat strain to represent the derivation and relatedness of the biosamples in the database. Work is underway to expand the quantitative phenotype dataset for chinchilla.

In the case of dog and pig, the Online Mendelian Inheritance in Animals database (OMIA, Sydney School of Veterinary Science, http://omia.org/) (52) has manually curated disease/phenotype annotations for genes in these species. To supply our users with this valuable, expert-reviewed information, RGD has instituted a pipeline to import these annotations and associate them with RGD’s corresponding gene records.

In each case, species have been selected and continue to be added on the basis of their status as established models for diseases of interest to RGD users. In addition, for species such as chinchilla and squirrel that are just moving into the realm of genomics, and for which there is little species-specific data available, their integration with more highly studied species can help to inform research going forward. Similarly, as the amount of whole genome sequence increases across all species, having access to information about variants in other species and demonstrated or predicted associations between those variants (and/or the genes and genomic regions in which they fall) and specific diseases or phenotypes can provide valuable insights to be used, for example, to narrow or prioritize a list of genes or gene variants in the species of interest to ones predicted to most likely be of interest based on data in other species.

### Data from the Alliance of Genome Resources

The need for precision models for human disease has prompted six model organism databases (MODs) and the Gene Ontology Consortium (GOC) (53,54) to collaborate to form the Alliance of Genome Resources (‘Alliance’, (55)). The founding members of the Alliance include RGD, MGI, ZFIN (*Danio rerio*) (56), FlyBase (*Drosophila melanogaster*) (57), WormBase (*Caenorhabditis elegans*) (58), and SGD (*Saccharomyces cerevisiae*) (59), in addition to the GOC. The Alliance integrates the output from these incredibly diverse research communities, making it a rich source of cross-species data. RGD provides access to this valuable data source through the link in the footer of most RGD pages and gene-specific links on all RGD rat, mouse and human gene pages. In addition, RGD has added the Alliance ortholog set to our database and provides the list of Alliance orthologs on applicable gene pages with links to the corresponding Alliance gene pages (Supplementary Figure S1, https://rgd.mcw.edu/rgdweb/report/gene/main.html?id=2077), facilitating access to additional types of data and additional models.

For a number of years, RGD has provided human-readable gene descriptions based on the associated ontology annotations for that gene and/or its orthologs. The algorithm used to produce these descriptions prioritized the annotations used for the descriptions based on whether the evidence for each annotation was experimental in the species of that gene, was experimental in another species or had been predicted informatically, and then selected three terms to be included in the description for that vocabulary. More recently, however, the Alliance has developed a more sophisticated algorithm for creating automated ontology annotation-based gene descriptions. This algorithm not only prioritizes annotations in much the same way that RGD’s procedure used, but also leverages the hierarchical structure of these vocabularies to find a lowest common parent (LCP) for cases where there are multiple annotations from the same branch of an ontology. In addition, when there are still too many terms to be able to show them all, it further prioritizes the LCP terms by taking into account the information content of the terms. RGD now imports these improved gene descriptions from the Alliance and applies them to the corresponding RGD gene records. In the case of the Pathway and ChEBI (used for gene-chemical interactions) ontologies, which are not used in the Alliance, RGD adds additional sentences for this data to the imported descriptions, giving users a complete at-a-glance picture of what is known about their gene of interest.

### Protein data

RGD is actively expanding the information presented that relates to proteins and other gene products. Protein sequences and links to additional information at other databases have been available from RGD gene pages almost from the beginning. In 2016, RGD began importing data for protein-protein interactions from the IMEx Consortium (21). The initial dataset included >150 000 interactions for rat, mouse and human. Recent updates also include interaction data for dog and pig. Work is nearly complete to also include data from the BioGrid database which almost doubles the number of interactions. BioGrid data from the Alliance that has already been QC’ed and matched to Alliance genes for rat, mouse and human is imported, simplifying the import process and capitalizing on work that has already been done at the Alliance. RGD’s interaction data can be queried, viewed and downloaded using the InterViewer tool ((60), and described below).

### Genome information pages

With the expansion of data types and species, RGD is working to present the data in both cross-species and species-focused ways. For instance, RGD now presents Genome Information pages for each species (https://rgd.mcw.edu/rgdweb/report/genomeInformation/genomeInformation.html, Supplementary Figure S2). Each page gives an integrated view of the information available about that species' genome sequence such as sequence statistics (the designation of the current assembly, total number of base pairs, total sequence length, total gap length, etc.), counts of the number of genes by type, and counts of genes with orthologs in other RGD species. For species with genomes that are assembled to the level of chromosomes, chromosome-specific pages are provided with more information about that chromosome and the data associated with it, including the number of genes on that chromosome annotated to each category of disease. Gene density plots are provided for each chromosome. Links are provided to additional information at NCBI, Ensembl and UCSC, as well as links to the sequence for each chromosome. Dropdown lists at the top of each genome information page facilitate navigation between species and between assemblies.

### Expanded programmatic access to RGD data

RGD’s FTP site has been reorganized to give researchers species-focused access to their datasets of interest. The data for each organism has been consolidated into a species-specific directory, so rat researchers, for instance, can go to the ‘RAT’ data release directory (ftp://ftp.rgd.mcw.edu/pub/data_release/RAT/) to access data for rat genes, QTLs, strains, markers, etc. as well as all protein-protein interaction data where one or both of the interactors are rat.

As a complement to the bulk download options in the RGD FTP site, REST APIs provide programmatic access to much of RGD’s data (https://rest.rgd.mcw.edu/rgdws/swagger-ui.html). Built on the Swagger framework, RGD’s APIs conform to the OpenAPI Specification.

## NEW TOOLS

### Tools for creating and analyzing gene/object lists, phenotype investigation and literature search

In recent years, RGD has developed a suite of tools to easily create and analyze a list of genes. These can be accessed from the ‘Analysis & Visualization’ link in the RGD front page menu. Tools that are specifically designed to help users analyze a list of genes appear in the RGD Toolbox (see below). In addition to the Gene Annotator (GA Tool) and Variant Visualizer described elsewhere (60,61), RGD’s toolbox includes the Online List Generator and Analyzer (OLGA) tool for composing a gene list based on a variety of criteria, the InterViewer for visualizing protein-protein interactions for lists of genes of interest, and a JBrowse iteration for each species, upgraded from GBrowse. These tools have been detailed previously (60,61), and are briefly described here. RGD’s newest tools include the Multi Ontology Enrichment Tool (MOET) for calculating term enrichment for five ontologies across species, the Gene and Ortholog Location Finder (GOLF) tool for obtaining a list of orthologs with their positions for an input list of genes, the Phenominer Expected Ranges tool to view strain-specific reference ranges for phenotype measurements under control conditions, and OntoMate, a text-mining tool for retrieving abstracts of interest; these four new tools are characterized in more detail below.

#### RGD’s toolbox

As the number of query and analysis tools has grown, it has concurrently increased the number of options of ‘where to go from here’ when a user creates or accesses a list of genes or other data objects. To help users more easily find and submit data to analysis tools, RGD has developed a ‘toolbox’ as a navigational aid. The toolbox provides simplified access to many of the same tools listed in the ‘Analysis & Visualization’ dropdown menu in the RGD page headers and on the ‘Analysis & Visualization’ landing page, and is accessible using the ‘Analyze’ tool icon which appears in multiple places on the site, including at the top of gene pages, on the ontology report pages, above the ‘Genes in Region’ table on QTL report pages, etc. The specific tools which appear in the toolbox at a particular point are species- and datatype-specific. Clicking the toolbox icon or link in a location that shows a list of genes will automatically send that list for analysis to whatever tool the user selects.

#### OLGA—Online List Generator and Analyzer

RGD’s Online List Generator and Analyzer (OLGA, https://rgd.mcw.edu/rgdweb/generator/list.html) tool is an advanced search tool which gives users the functionality to create lists based on a variety of criteria and combine them in multiple ways to assemble their own custom list of genes, QTLs or strains (60,61). For instance, for genes users can identify lists of genes that are annotated to a specified term (or its children) in one of five of the ontologies used at RGD (Disease, Pathway, Mammalian Phenotype, GO Biological Process, GO Molecular Function, GO Cellular Component, or ChEBI) or that map within a specified genomic region or QTL region, or upload a list of gene symbols. In each case, the list can then be combined (via a union, intersection or subtraction) with other lists of the same type generated in a similar fashion. At each step of the process, additional lists can be generated and combined with the current result, or ‘Analyze Result Set’ can be selected to access the RGD Toolbox.

#### InterViewer—protein–protein interaction viewer

RGD’s InterViewer (https://rgd.mcw.edu/rgdweb/cytoscape/query.html) is a Cytoscape-based tool for visualizing protein-protein interactions. Built upon the foundation of interaction data which RGD imports from the IMEx Consortium (21), InterViewer accepts UniProtKB protein IDs, gene RGD IDs and gene symbols. Data is currently available for rat, human, mouse, dog and pig. For users interested in viewing all rat interactions or downloading all of the interaction data for all applicable RGD species, links are provided on the InterViewer landing page to access a page listing all of the rat binary interactions in the former case, and the interaction FTP files in the latter case. To view and/or download a limited number of interactions, submit one or more identifiers to the tool. The result page [Supplementary Figure S3, and (60,61)] shows both a Cytoscape graph and a tabular list of the binary interactions for the input list of proteins, or the proteins associated with an input list of genes. The downloadable table below the graph gives specific information about each interaction displayed. When the input list would result in too many interactions to be displayed in a graph, InterViewer provides a link to download that list of interactions.

#### Upgrade from GBrowse to JBrowse and implementation for all RGD species

To support the incorporation of large datasets such as RNA-Seq data and variation tracks from whole genome sequencing, RGD has moved away from using the ‘Generic Genome Browser’ GBrowse (22) in favor of the faster and more dynamic Javascript-based JBrowse genome browser (23). RGD currently maintains instances of JBrowse for three assemblies each for rat and human, two each for mouse and pig, and one each for bonobo, chinchilla, dog and squirrel. All instances of JBrowse at RGD include tracks for the reference sequence, genes and transcripts, as well as a combined track that shows each gene with a group of its transcripts. In addition, the rat, mouse and human JBrowse instances have additional tracks such as strain and strain-specific variant tracks for rat, and tracks for QTLs, disease-associated objects, ChEBI-associated genes and markers for all three species. For more information about RGD’s JBrowse, see (60,61).

#### MOET—Multi Ontology Enrichment Tool

**Figure 2.**
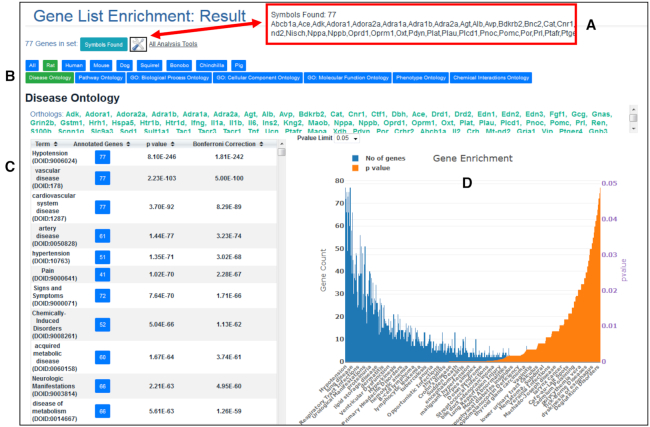
MOET: the Multi Ontology Enrichment Tool Result Page. Seventy seven rat genes annotated to hypotension in RGD were submitted to the MOET tool. The result page shows the number of genes that matched the input list of symbols and when the ‘Symbols Found’ button is clicked, the popup shows the list of gene symbols (**A**). This same list of genes can be submitted to other tools at RGD using the ‘All Analysis Tools’ link. Click on the tabs across the top of the page to switch the display between species and/or between ontologies. Here, the default selections of ‘Rat’ and ‘Disease Ontology’ have been made (**B**). The table on the left side of the display shows the enriched terms with the number of genes annotated to each term, its *P*-value and its Bonferroni corrected *P*-value. The default is to show terms sorted by *P*-value but options are given to sort on any of the columns in the table (**C**). The graph on the right side of the display shows the number of genes annotated to each term in blue and the corresponding *P*-values in orange. The *P*-values displayed in the graph are limited to 0.05 or less by default but a dropdown at the top of the graph allows the user to change this value (**D**).

RGD’s Multi Ontology Enrichment Tool (MOET) takes a list of genes or a region of interest, such as a QTL region, and calculates term enrichment values, that is, the statistical probability that a specific ontology term is more highly represented in the annotations for a given list of genes than would be expected by chance given the annotations for all genes for that species, for any or all of five ontologies—Disease, Pathway, GO, Phenotype and ChEBI. Enrichment is calculated for the input species and for the orthologs of the input gene list in the rest of RGD’s species. The result page (Figure 2) shows the list of genes in RGD that match the input criteria. Buttons give one-click access to the results for other species and/or other ontologies. A result table gives the list of enriched terms with the p-value and Bonferroni-corrected p-value for each. The list can be sorted on any column. A button in the ‘Annotated Genes’ column of the table pulls up the list of genes from the input list that are annotated to the corresponding term or any of its children, with the specific term to which the gene is annotated. The ‘Explore this Gene Set’ link in the popup sends that subset of genes back to the MOET tool for reanalysis of the ontology enrichment for those genes. The result page also shows a graph of the enrichment results as the number of genes annotated to a specific term in blue and the p-value for that term in orange. The ‘All Analysis Tools’ link at the top of the page opens the RGD toolbox. Select a tool from the toolbox to submit the list of genes input for the MOET analysis (or the subset submitted for the reanalysis) to any of RGD’s other tools.

#### GOLF—Gene and Ortholog Location Finder

RGD’s newest tool is GOLF, the Gene and Ortholog Location Finder (Supplementary Figure S4). Select an input species and genome assembly, enter a list of gene symbols or a region of interest, and select an output species and genome assembly. The tool will return the genes from the input list with their genomic positions on the specified assembly and the list of orthologs in the output species with their positions on the requested output assembly. The tool also allows comparisons between assemblies for a single species. For instance, to compare the genomic positions for a list of genes between the rat Rnor5.0 and Rnor6.0 assemblies, choose rat for both input and output and specify those two assemblies. The tool gives the positions of each gene from the list on both assemblies. Download the results or send the input or output list of genes to other analysis tools in RGD using the prominent links above the result table.

#### PhenoMiner Expected Ranges tool

The Expected Ranges tool (Supplementary Figure S5) allows users to visualize the range of a selected phenotype measurement among control data for inbred rat strains across several studies. Using curated data from the PhenoMiner tool, rat substrains were clustered/aggregated into strain groups, and where four or more experiments existed for a given strain group and measurement, a standard meta-analysis was performed to determine the expected range of values for that measurement in control rats of that strain (62). The Expected Ranges tool groups measurements according to the trait each measures using the Vertebrate Trait Ontology. For instance, ‘circulatory system physiology trait’ includes several blood pressure measurements and heart rate. For each measurement, the PhenoMiner Expected Ranges result page shows a graph of the ranges for all applicable strain groups. Where enough data is available, the measurement values have been stratified by age, sex or, for some measurements such as blood pressure, method. Where this has occurred, in addition to the range calculated for the full set of data, expected ranges have been calculated for subsets of the data, e.g. for measurements of male rats only or female rats only. These stratified values are also displayed in the graph. A panel above the graph gives options for filtering the results based on strain, age, sex and where applicable, the method. A table below the graph shows additional information for each range value including the strains in the group for that calculation and the mean, standard deviation, low value and high value of the range. A link in the last column of the table takes users to the PhenoMiner display for the specific records that were used for the standard range calculation.

In addition to querying by phenotype measurements, the calculated expected ranges can also be visualized by strain groups. For each strain group, the result page shows all of the measurements for which there is an expected range for that group. For sequenced strains, the number of damaging variants for each assembly is shown in a table on the expected ranges page for that strain group. The counts link to a downloadable list of the variants with their positions, reference and variant nucleotides, and gene symbols. Linking phenotype values and genomic variations using the Expected Ranges tool will provide a useful approach to understanding the complexities of physiological genomics.

#### OntoMate literature ‘smart search’

RGD’s OntoMate was developed as an ontology-driven custom text-mining software tool originally designed to be tightly integrated with RGD’s curation software (63). OntoMate's backend tools analyze the text and extract information from publications in order to enrich processed articles with semantic tags, including all gene symbols in the RGD database, mutations, all of the species found in the NCBI taxonomy, and terms from eleven ontologies used for curation. The proven usefulness of this tool in helping curators more efficiently locate papers that meet their criteria with fewer irrelevant results prompted RGD to extend and adapt the software, creating a user interface giving researchers access to OntoMate as a literature search engine on RGD’s public website.

**Figure 3.**
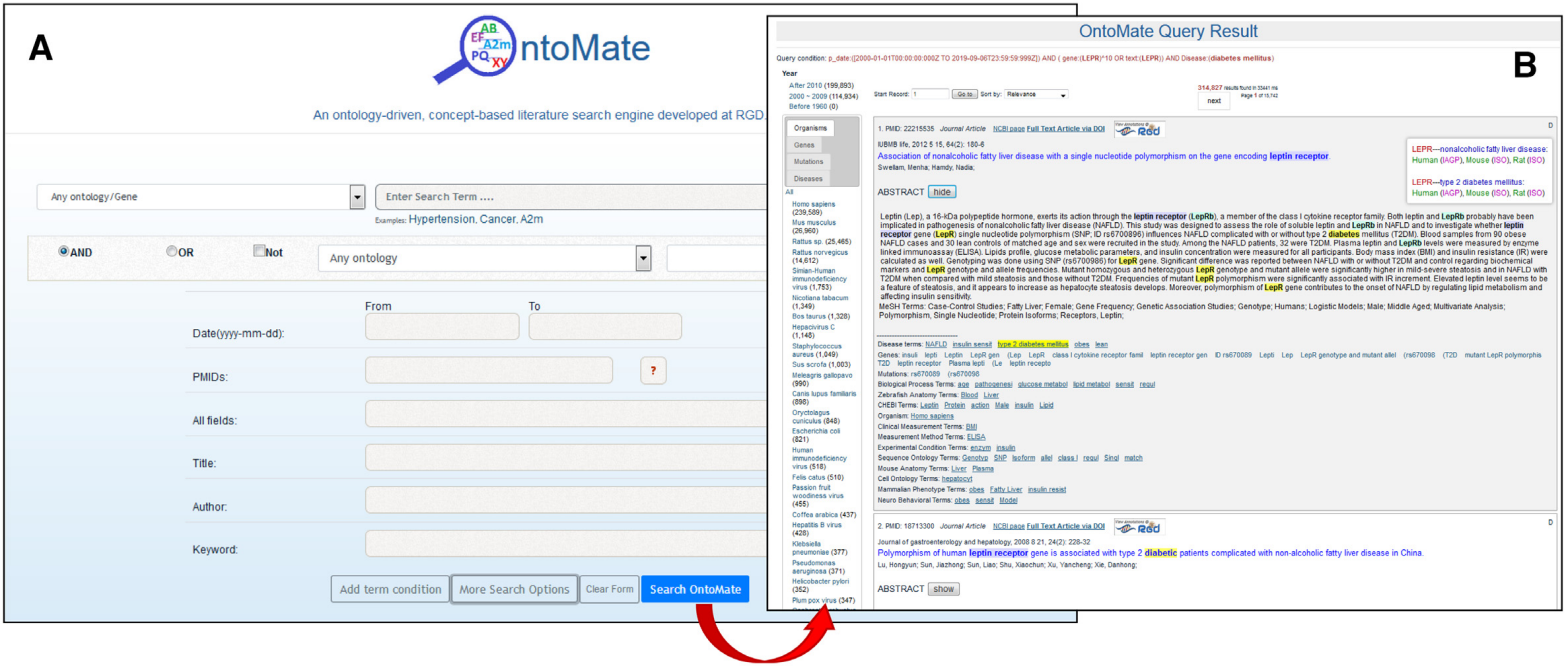
The OntoMate text mining-based literature search tool. The OntoMate tool uses text mining and natural language processing to find and tag abstracts with ontology terms, gene symbols and names, species terms and mutation designations. The search interface (**A**) gives options to specify any of these, as well as searching or filtering the results by date, PubMed ID, abstract title, author or keyword(s). The Query Result page (**B**) shows each abstract with its tags and provides the user with additional filtering options. See Supplementary Figure S6 for a more complete description.

The OntoMate tool is accessed on the RGD home page from the ‘Analysis & Visualization’ pulldown menu of tools. To start a search, ‘Gene’ or an ontology category is specified in the first box and the gene name, symbol or term is entered into the second box, which has an autocomplete feature (Figure 3, Supplementary Figure S6). The search can be restricted using ‘Add term condition’ to add more terms or genes with selected Boolean relationships. Additional filters may be used with ‘More Search Options’, which allows searching/filtering by date, PubMed ID(s), title, author or PubMed keyword, or entering a term to search across all fields in the data store. The search is then executed by clicking ‘Search OntoMate’. This retrieves a list of matching abstracts, with the Boolean search used shown at the top left. The total number of abstracts found is shown at the top right, with the number of pages and a ‘next’ button to advance to the next page. To the left is a function to go to a specific abstract number, and the ability to sort the results by a pulldown menu of options – relevance, publication date or PubMed ID. A series of filters are available in a column at the left, allowing selection of year ranges, or specific terms in the categories ‘organisms’, ‘genes’, ‘mutations’ or ‘diseases’. For each filtered category, the number of abstracts is given in parentheses. Clicking the category term brings up that list of abstracts.

For each abstract, clicking ‘show’ next to ‘ABSTRACT’ displays the abstract text, with searched terms and searched gene names and symbols highlighted. At the top of each abstract, clicking ‘NCBI page’ goes to the abstract in PubMed, while clicking ‘Full Text Article via DOI’ or ‘Free PMC Article’ where available goes to the full paper. The presence of an RGD icon to the right indicates that there are manual annotations associated with the publication. Clicking this goes to a report page indicating all the terms and objects annotated to the paper. The letter(s) in the upper right corner of the abstract box indicate what kind of annotations exist, and mousing over them displays them in more detail. Mousing over the linked terms in listed categories below the abstract highlights them within the abstract text; clicking them goes to their respective ontology term report or informational pages.

## DISEASE PORTALS: NEW DISEASE CATEGORIES, UPDATED TECHNOLOGY

**Figure 4.**
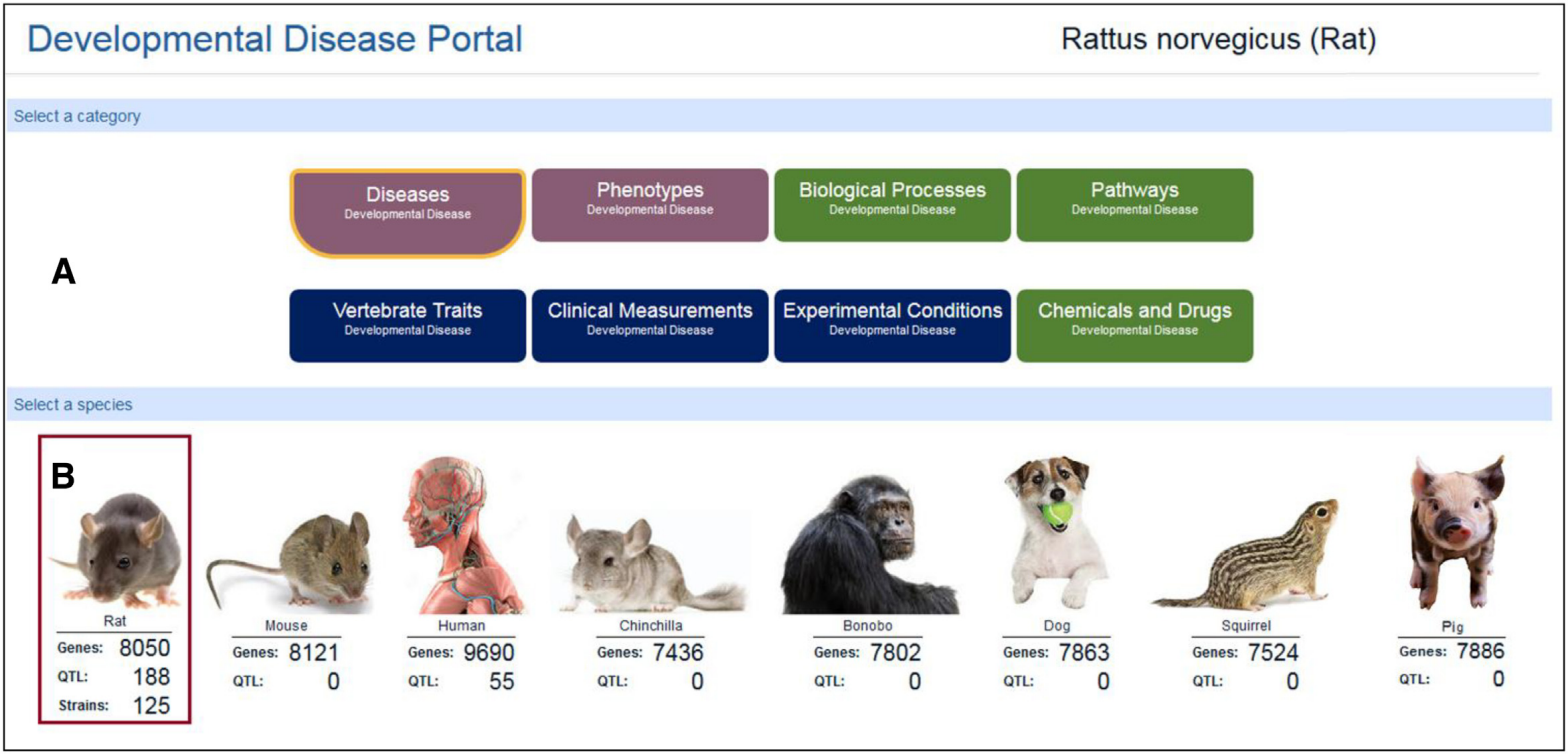
The Developmental Disease Portal. The top of the page in each disease portal shows the name of the portal and indicates which species' data is being shown. The data showing in the portal can be changed by selecting a different data type, e.g. Phenotypes or Pathways versus Disease, using the buttons in the top panel (**A**), or a different species by selecting a picture in the second panel (**B**). For more information and a full view of a portal page, see Supplementary Figure S7.

The Disease Portals at RGD are an integrated resource for exploring data associated with specific disease categories. Recently, RGD’s portals were completely redesigned to increase both the functionality and the incorporated data. The portals have been expanded to twelve disease areas populated with manual annotations created at RGD and imported annotations from other data sources. In addition, each portal now includes data for all eight of RGD’s species (Figure 4, Supplementary Figure S7). Specific disease information can be accessed by choosing an ontology or a species, and the selection can be modified during data queries. The updated portals house a customized ontology browser. For disease, this browser displays terms related to the disease category covered by the portal which have been used for annotations at RGD. Terms displayed for other ontologies are derived from annotations assigned to disease-related genes, QTLs and/or strains. Annotated genome objects can be viewed on the Ontology Report page accessed by clicking on the ‘A’ icon next to the ontology term, as well as in the portal itself in the display boxes below the ontology browser. The data in each of these lists can be downloaded for further analysis. RGD’s integrated Genome Viewer shows the positions of the genes, QTLs and strains listed in the boxes directly above it against the full set of chromosomes for the selected species. The object lists and the GViewer display will immediately reflect changes to the selection made in the ontology browser. Below the GViewer is a new embedded enrichment tool. Users can select any of up to seven ontologies to view a MOET-type display of over-represented terms in annotations for the currently displayed gene list.

## NEW AND ONGOING COLLABORATIONS WITH THE LARGER BIOINFORMATICS COMMUNITY

In addition to expanding the available resources within RGD for the benefit of our users, RGD is committed to collaborating with the larger research and bioinformatics community. As mentioned previously, RGD is one of the founding members of the Alliance of Genome Resources (the Alliance, (55)) whose stated mission is ‘to develop and maintain sustainable genome information resources that facilitate the use of diverse model organisms in understanding the genetic and genomic basis of human biology, health and disease. This understanding is fundamental for advancing genome biology research and for translating human genome data into clinical utility.’ In addition to contributing data to the Alliance, RGD curators and developers actively participate in discussions and work to develop global data standards in order to unify the diverse data models that exist across the member MODs.

RGD also collaborates with other databases for data sharing, data QC and validation, and ontology development. In this regard, RGD maintains an ongoing collaboration with the HUGO Gene Nomenclature Committee (HGNC) at the European Bioinformatics Institute and MGI at The Jackson Laboratory to coordinate gene nomenclature for orthologs across the species, and with MGI to ensure that strains and alleles are named correctly. The three groups meet regularly to discuss issues and develop standards which can be used for nomenclature, not only in these three species but across all vertebrate species (64).

From its inception, RGD has had a data sharing collaboration with the National Center for Bioinformatics (NCBI). RGD downloads gene, transcript, protein, and genomic assembly information from NCBI, while NCBI takes nomenclature, summary/descriptions, Gene Ontology (GO) annotations and reference associations for genes, as well as QTL records from RGD. In addition, RGD and NCBI both share data QC and validation information, periodically exchanging information about records which should be merged or deprecated at either or both of the repositories. More recently, RGD has begun to also import gene data from Ensembl, and Ensembl uploads nomenclature and ontology annotations from RGD. RGD also collaborates with the Comparative Toxicogenomics Database (CTD) (65), importing gene-chemical interaction data and disease annotations from CTD and providing CTD with information about specific publications which contain gene-chemical interaction data that CTD can curate, and more recently has begun generating in-house gene-chemical interaction annotations.

Finally, RGD actively participates in ontology development for the Human Disease Ontology (DO) (66) and GO (54), requesting new terms, edits to existing terms and updates to the structure of the ontologies (i.e. new term-term relationships). RGD attends GO Consortium meetings regularly and meets with other developers of DO to discuss issues, improvements and priorities.

## CONCLUSION

At twenty, RGD has matured from a simple data repository into a cross-species knowledgebase providing structured, standardized data for multiple mammalian species, tools to facilitate the analysis of that data, and results from analyses that RGD has done which researchers can leverage, both to inform their own calculations and experimentation, and as input for further downstream analyses. In the past 12 months, RGD has had 215 575 users and 789 226 page views. A total of 76 913 files were downloaded from the RGD FTP site. The number of users has steadily increased over the past ten years with a noticeable increase in the past year, with totals for the years between 2010 and 2019 of over a million users and six million page views (Supplementary Figure S8). Going forward, RGD will continue incorporating additional animal species, diversifying data types, and designing and building new, innovative search and analysis tools to support research in a wide variety of disciplines and help researchers pinpoint the best precision model to explore their disease, phenotype or physiological question of interest.

## DATA AVAILABILITY

RGD’s data and tools are available on the RGD website at https://rgd.mcw.edu. Data can be downloaded from the RGD FTP site at ftp://ftp.rgd.mcw.edu/pub/data_release/ or using the REST APIs at https://rest.rgd.mcw.edu/rgdws/swagger-ui.html.

## Supplementary Material

gkz1041_Supplemental_File
