# Supplementary material for: The Year of the Rat: The Rat Genome Database at 20: a multi-species knowledgebase and analysis platform
Source: Nucleic Acids Res. 2019 Nov 12;48(D1):D731–42. doi: 10.1093/nar/gkz1041 (PMC7145519; doi:10.1093/nar/gkz1041)
Supplement: gkz1041_Supplemental_File [file gkz1041_supplemental_file.pdf]

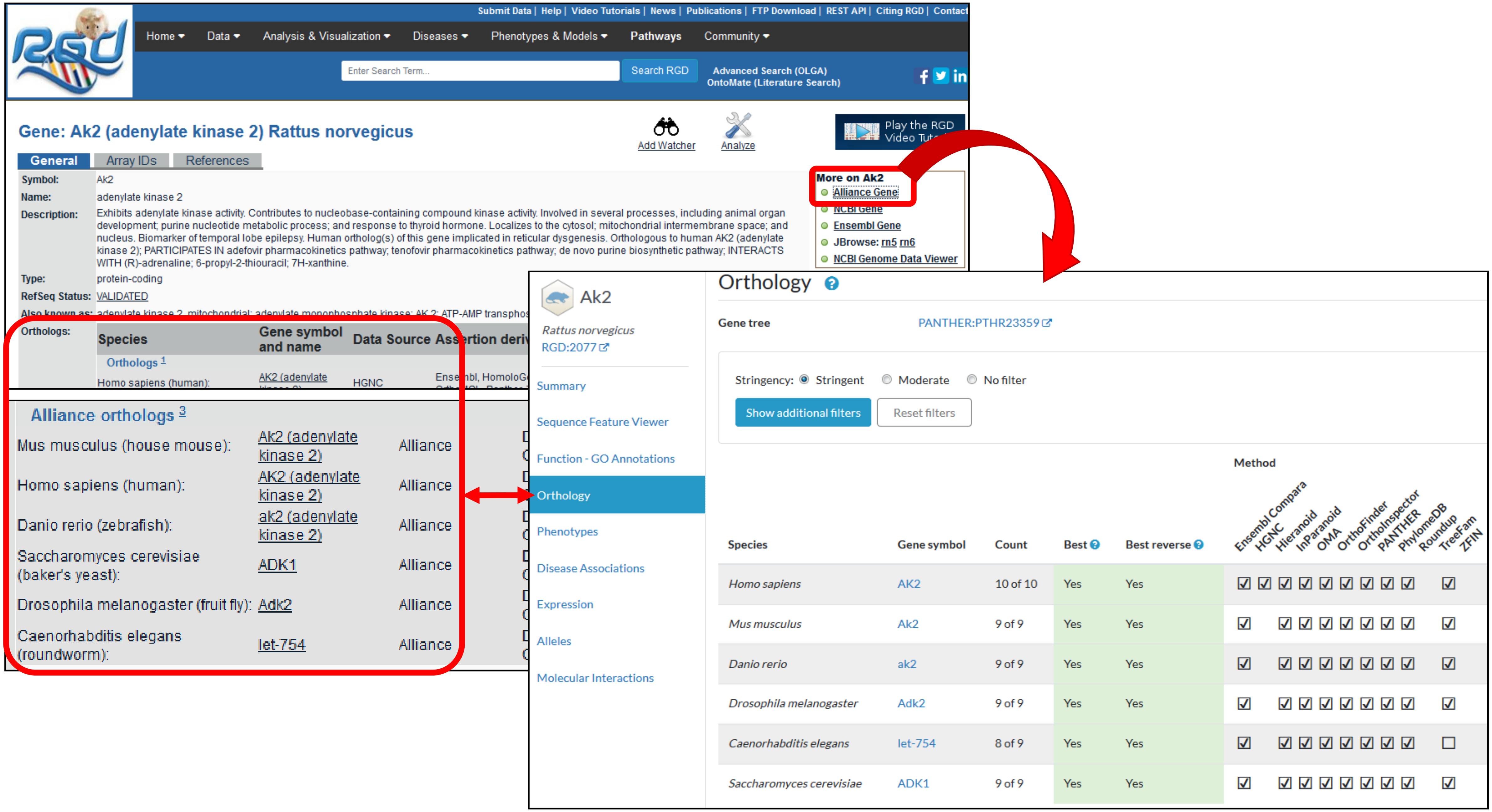

Supplementary Figure S1. Alliance orthologs. In the ortholog section of RGD gene report pages, clicking "more info…" expands the display of orthologs for RGD's seven other species to show the ortholog assignments for any or all of six species imported from the Alliance. Each symbol links to the orthologous gene on the Alliance website. A link at the top of each of RGD's rat, mouse and human gene pages also links to the Alliance page for that gene.

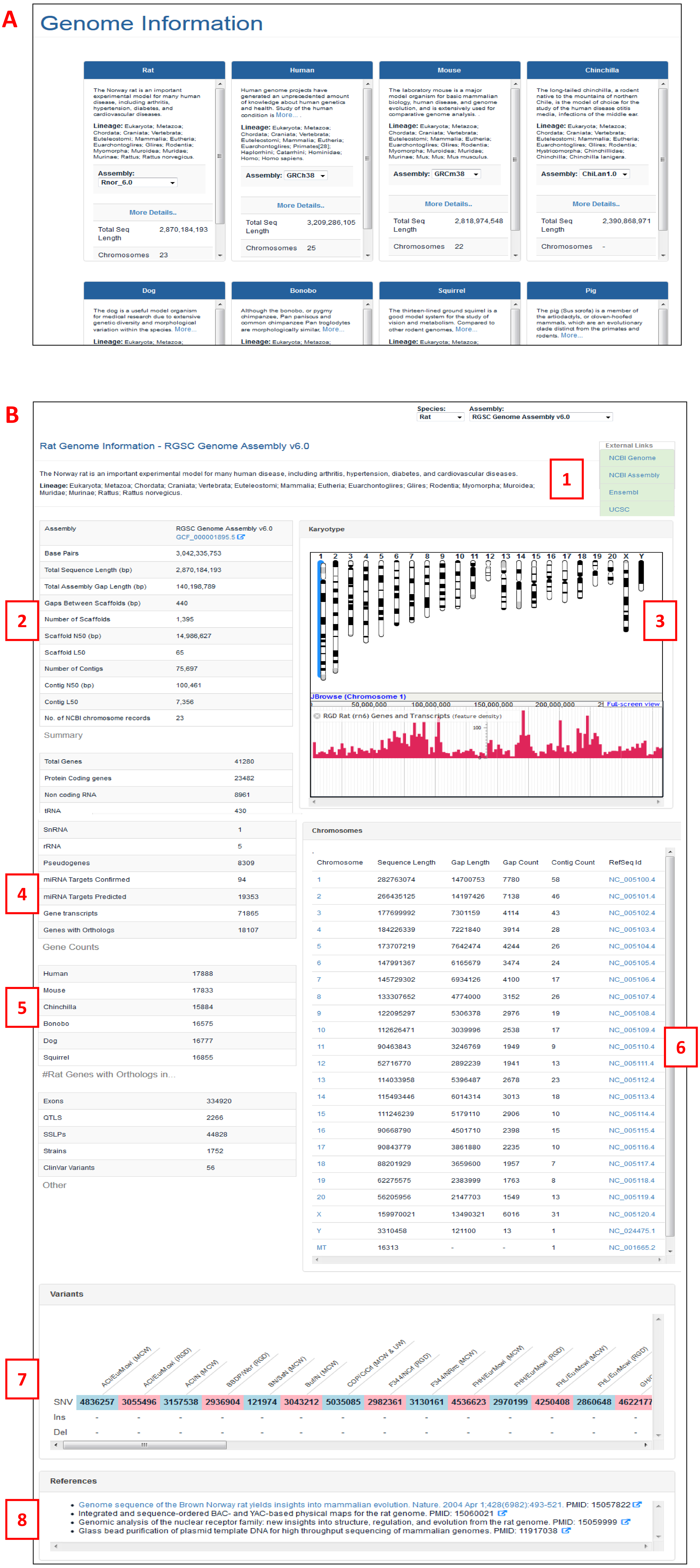
A
Genome Information
Rat
Human
Mouse
Chinchilla
Assembly: Rnor_6.0
Assembly: GRCh38
Assembly: GRCm38
Assembly: ChiLan1.0
More Details..
Total Seq Length 2,870,184,193
Total Seq Length 3,209,286,105
Total Seq Length 2,818,974,548
Total Seq Length 2,390,868,971
Chromosomes 23
Chromosomes 25
Chromosomes 22
Chromosomes -
Dog
Bonobo
Squirrel
Pig
B
Species: Rat
Assembly: RGSC Genome Assembly v6.0
Rat Genome Information - RGSC Genome Assembly v6.0
The Norway rat is an important experimental model for many human disease, including arthritis, hypertension, diabetes, and cardiovascular diseases.
Lineage: Eukaryota; Metazoa; Chordata; Craniata; Vertebrata; Euteleostomi; Mammalia; Eutheria; Euarchontoglires; Glires; Rodentia; Myomorpha; Muroidea; Muridae; Murinae; Rattus; Rattus norvegicus.
External Links
NCBI Genome
NCBI Assembly
Ensembl
UCSC
1
2
3
4
5
6
7
8
Assembly RGSC Genome Assembly v6.0 GCF_000001895.5
Base Pairs 3,042,335,753
Total Sequence Length (bp) 2,870,184,193
Total Assembly Gap Length (bp) 140,198,789
Gaps Between Scaffolds (bp) 440
Number of Scaffolds 1,395
Scaffold N50 (bp) 14,986,627
Scaffold L50 65
Number of Contigs 75,697
Contig N50 (bp) 100,461
Contig L50 7,356
No. of NCBI chromosome records 23
Summary
Total Genes 41280
Protein Coding genes 23482
Non coding RNA 8961
tRNA 430
SnRNA 1
rRNA 5
Pseudogenes 8309
miRNA Targets Confirmed 94
miRNA Targets Predicted 19353
Gene transcripts 71865
Genes with Orthologs 18107
Gene Counts
Human 17888
Mouse 17833
Chinchilla 15884
Bonobo 16575
Dog 16777
Squirrel 16855
#Rat Genes with Orthologs in...
Exons 334920
QTLS 2266
SSLPs 44828
Strains 1752
ClinVar Variants 56
Other
Karyotype
JBrowse (Chromosome 1)
RGD Rat (rn6) Genes and Transcripts (feature density)
Full-screen view
Chromosomes
Chromosome Sequence Length Gap Length Gap Count Contig Count RefSeq Id
1 282763074 14700753 7780 58 NC_005100.4
2 266435125 14197426 7138 46 NC_005101.4
3 177699992 7301159 4114 43 NC_005102.4
4 184226339 7221840 3914 28 NC_005103.4
5 173707219 7642474 4244 26 NC_005104.4
6 147991367 6165679 3474 24 NC_005105.4
7 145729302 6934126 4100 17 NC_005106.4
8 133307652 4774000 3152 26 NC_005107.4
9 122095297 5306378 2976 19 NC_005108.4
10 112626471 3039996 2538 17 NC_005109.4
11 90463843 3246769 1949 9 NC_005110.4
12 52716770 2892239 1941 13 NC_005111.4
13 114033958 5396487 2678 23 NC_005112.4
14 115493446 6014314 3013 18 NC_005113.4
15 111246239 5179110 2906 10 NC_005114.4
16 90668790 4501710 2398 15 NC_005115.4
17 90843779 3861880 2235 10 NC_005116.4
18 88201929 3659600 1957 7 NC_005117.4
19 62275575 2383999 1763 8 NC_005118.4
20 56205956 2147703 1549 13 NC_005119.4
X 159970021 13490321 6016 31 NC_005120.4
Y 3310458 121100 13 1 NC_024475.1
MT 16313 - - 1 NC_001665.2
Variants
ACI/EurMcwi (MCW)
ACI/EurMcwi (RGD)
ACI/N (MCW)
BBDP/Wor (RGD)
BN/SsN (MCW)
Buf/N (MCW)
COP/CrCrl (MCW & UW)
F344/NCrl (RGD)
F344/NRrrc (MCW)
FHH/EurMcwi (MCW)
FHH/EurMcwi (RGD)
FHL/EurMcwi (MCW)
FHL/EurMcwi (RGD)
SNV 4836257 3055496 3157538 2936904 121974 3043212 5035085 2982361 3130161 4536623 2970199 4250408 2860648 4622177
Ins
Del
References
Genome sequence of the Brown Norway rat yields insights into mammalian evolution. Nature. 2004 Apr 1;428(6982):493-521. PMID: 15057822
Integrated and sequence-ordered BAC- and YAC-based physical maps for the rat genome. PMID: 15060021
Genomic analysis of the nuclear receptor family: new insights into structure, regulation, and evolution from the rat genome. PMID: 15059999
Glass bead purification of plasmid template DNA for high throughput sequencing of mammalian genomes. PMID: 11917038

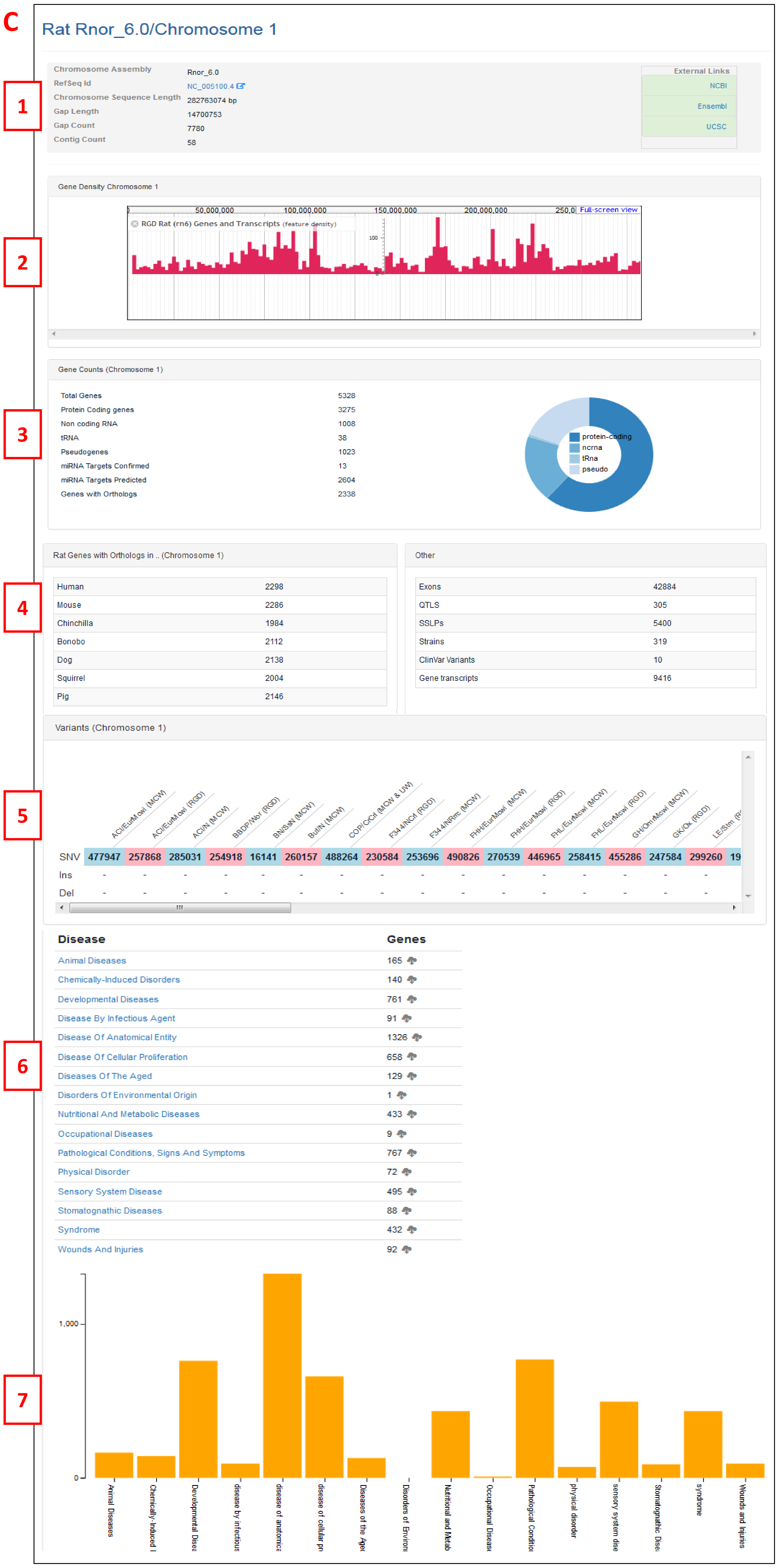

Supplementary Figure S2. The Genome Information Page. RGD displays Genome Information pages for each species in the database. Each panel contains a short summary of the research uses of each animal, the placement of the species in the NCBI taxonomy, a dropdown for selecting the assembly, and a list of statistics for that species' genome sequence (A). The main Genome Information page for rat Rnor 6.0 assembly (B) shows the summary of research uses and lineage, and provides links to NCBI, Ensembl and UCSC for that species (B1); statistics for the sequence and assembly with a link to the assembly record at NCBI (B2); karyotype view with chromosome 1 highlighted and an embedded view of the gene density histogram for that chromosome from JBrowse (B3); list of the number of genes by type, transcripts and genes with any ortholog in the rat genome (B4); counts of the number of rat genes that have an ortholog in each of RGD's other species (B5); information about each chromosome with a link to the RefSeq file for that chromosome (B6) - each chromosome number links to a detail page for that chromosome (C); counts of the total number of variants on that assembly for each sequenced rat strain (B7); links to seminal publications for the rat assembly (B8). The detail page for rat chromosome 1 (C) contains information about the chromosome with links to the chromosome 1 sequence and to the major databases (C1); an embedded view of the gene density histogram for chromosome 1 (C2); tabular and graphical view of the number of genes by type for chromosome 1 (C3); counts of the number of rat genes on chromosome 1 that have an ortholog in each of RGD's other species and counts of other objects found on chromosome 1 such as QTLs and mapped strains (C4); counts of the number of variants on chromosome 1 in Rnor 6.0 for each sequenced rat strain (C5); a tabular (C6) and graphical (C7) view of the number of genes on chromosome 1 with annotations to each of the major disease categories in the disease ontology.

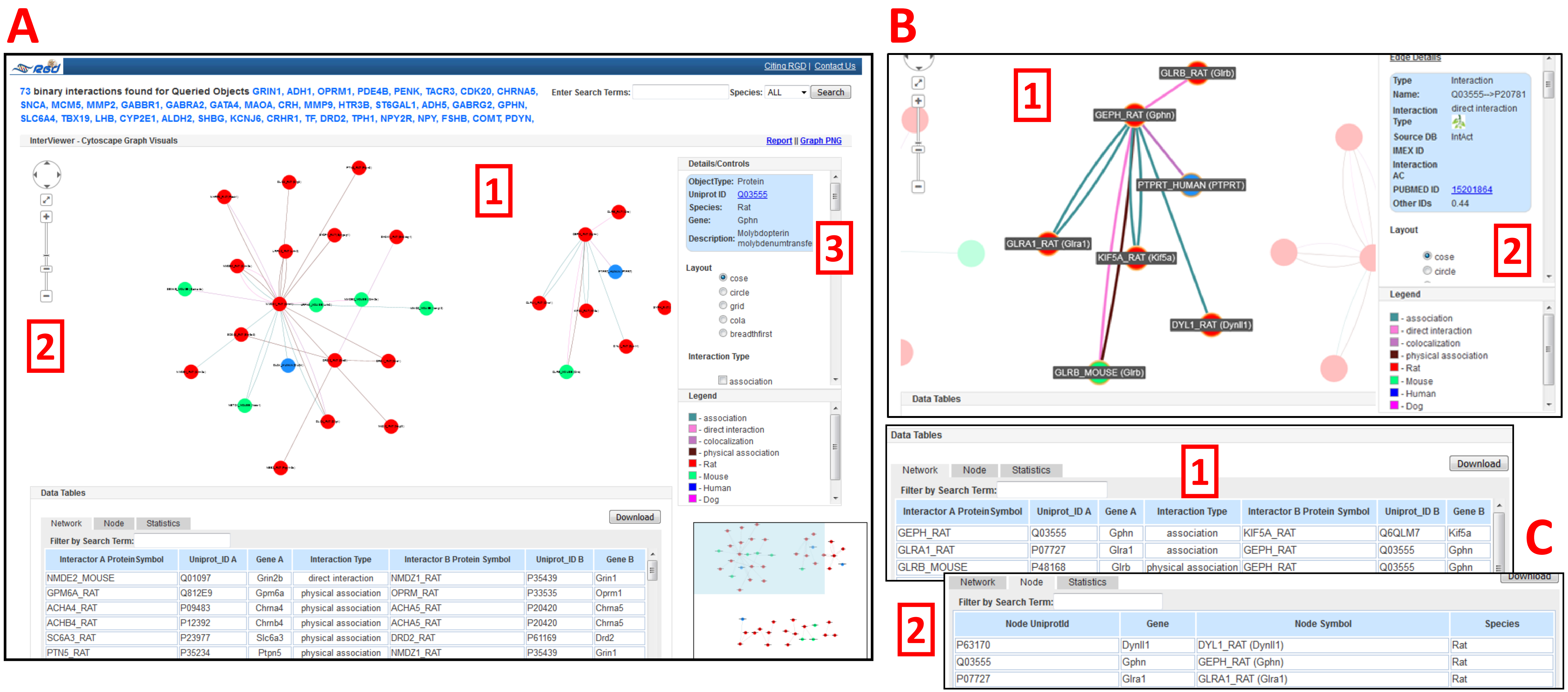

Supplementary Figure S3. The InterViewer tool. Result page (A); Cytoscape display of binary protein-protein interactions (A1); Zoom and directional display control (A2); scrollable panel containing information display for selected protein, alternative layout options, interaction type selector, species selector (A3); zoomed display showing a selected protein with its interactors (nodes) and highlighted interactions (edges) (B1); key showing the color of the edge line for each interaction type and the color of the node circle for each species (B2); the "Network" tab contains a downloadable tabular display of binary interaction data showing protein symbols and IDs and gene symbols for both interactors separated by a column showing the interaction type (C1); a nonredundant list under the "Node" tab displays information for each interactor in the network (C2).

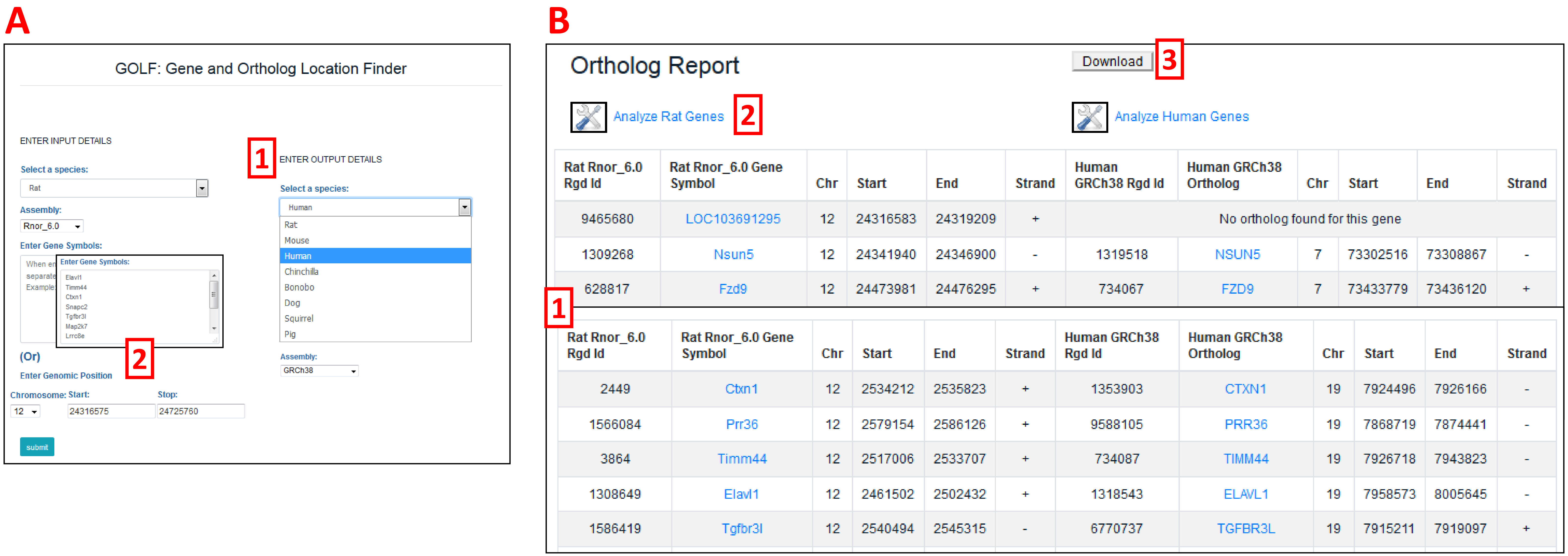

Supplementary Figure S4. The Gene and Ortholog Location Finder (GOLF) Tool. RGD's GOLF tool takes either a list of gene symbols or a genomic position for one species as input and returns the corresponding gene positions in the selected assembly and the list of orthologs with their positions in another species. In the GOLF query form (A), select an input species and assembly and an output species and assembly (A1). When the two species are different, GOLF gives the list of orthologs for the input genes. When the user selects the same species for both input and output, GOLF will return the list of the same genes in that species with positions in two different assemblies. Input for the query can be either a list of gene symbols, or a genomic position (i.e., chromosome, start and stop) (A2). The GOLF result page, i.e., the Ortholog Report (B), shows a table with the RGD ID, gene symbol, chromosome, start position, stop position and strand for each gene in the input list and each ortholog in the output species (B1). Column headers give both input and output species and assembly. In this example, in the top list the rat and human genes are on the same strand of the chromosome and the order of the genes on the chromosome in the two species is the same. In the bottom list, the genes are on opposite strands and the gene order is reversed. Links are provided to easily submit either result list to the toolbox for additional analysis (B2), and to download the full set of results (B3).

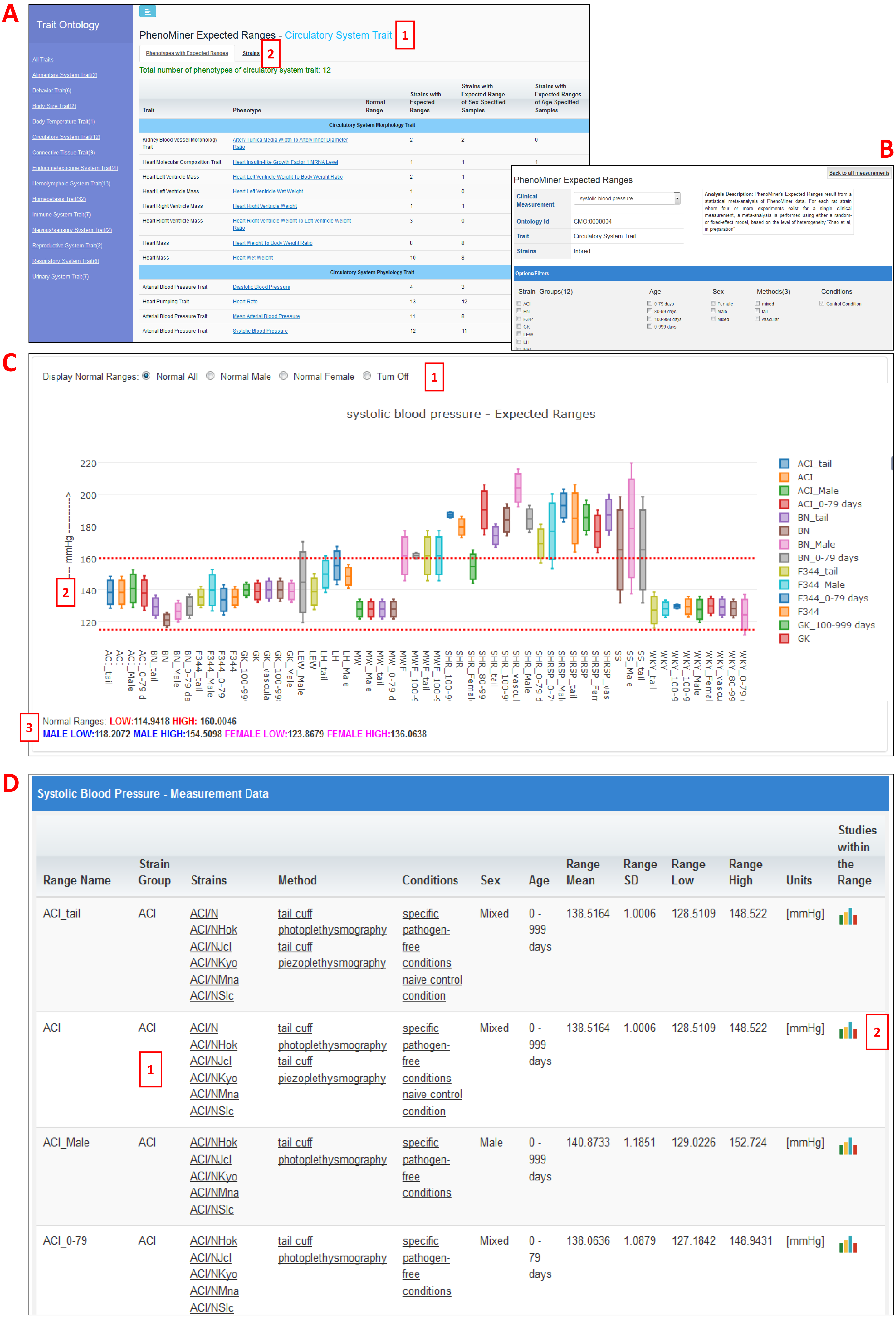
A
Trait Ontology
PhenoMiner Expected Ranges - Circulatory System Trait
1
Phenotypes with Expected Ranges
Strains
2
Total number of phenotypes of circulatory system trait: 12
Trait
Phenotype
Normal Range
Strains with Expected Ranges
Strains with Expected Range of Sex Specified Samples
Strains with Expected Ranges of Age Specified Samples
Circulatory System Morphology Trait
Kidney Blood Vessel Morphology Trait
Artery Tunica Media Width To Artery Inner Diameter Ratio
Heart Molecular Composition Trait
Heart Insulin-like Growth Factor 1 MRNA Level
Heart Left Ventricle Mass
Heart Left Ventricle Weight To Body Weight Ratio
Heart Left Ventricle Wet Weight
Heart Right Ventricle Mass
Heart Right Ventricle Weight
Heart Right Ventricle Weight To Left Ventricle Weight Ratio
Heart Mass
Heart Weight To Body Weight Ratio
Heart Wet Weight
Circulatory System Physiology Trait
Arterial Blood Pressure Trait
Diastolic Blood Pressure
Heart Pumping Trait
Heart Rate
Mean Arterial Blood Pressure
Systolic Blood Pressure
All Traits
Alimentary System Trait(2)
Behavior Trait(6)
Body Size Trait(2)
Body Temperature Trait(1)
Circulatory System Trait(12)
Connective Tissue Trait(9)
Endocrine/exocrine System Trait(4)
Hemolymphoid System Trait(13)
Homeostasis Trait(32)
Immune System Trait(7)
Nervous/sensory System Trait(2)
Reproductive System Trait(2)
Respiratory System Trait(6)
Urinary System Trait(7)
B
PhenoMiner Expected Ranges
Back to all measurements
Clinical Measurement
systolic blood pressure
Ontology Id
CMO:0000004
Trait
Circulatory System Trait
Strains
Inbred
Options/Filters
Strain_Groups(12)
Age
Sex
Methods(3)
Conditions
C
Display Normal Ranges: Normal All Normal Male Normal Female Turn Off
1
systolic blood pressure - Expected Ranges
mmHg
2
ACI_tail
ACI
ACI_Male
ACI_0-79 days
BN_tail
BN
BN_Male
BN_0-79 days
F344_tail
F344_Male
F344_0-79 days
F344
GK_100-999 days
GK
3
Normal Ranges: LOW:114.9418 HIGH: 160.0046
MALE LOW:118.2072 MALE HIGH:154.5098 FEMALE LOW:123.8679 FEMALE HIGH:136.0638
D
Systolic Blood Pressure - Measurement Data
Range Name
Strain Group
Strains
Method
Conditions
Sex
Age
Range Mean
Range SD
Range Low
Range High
Units
Studies within the Range
ACI_tail
ACI
ACI/N
ACI/NHok
ACI/NJcl
ACI/NKyo
ACI/NMna
ACI/NSlc
tail cuff photoplethysmography
tail cuff piezoplethysmography
specific pathogen-free conditions
naive control condition
Mixed
0 - 999 days
138.5164
1.0006
128.5109
148.522
[mmHg]
ACI
ACI
ACI/N
ACI/NHok
ACI/NJcl
ACI/NKyo
ACI/NMna
ACI/NSlc
tail cuff photoplethysmography
tail cuff piezoplethysmography
specific pathogen-free conditions
naive control condition
Mixed
0 - 999 days
138.5164
1.0006
128.5109
148.522
[mmHg]
1
2
ACI_Male
ACI
ACI/NHok
ACI/NJcl
ACI/NKyo
ACI/NMna
ACI/NSlc
tail cuff photoplethysmography
specific pathogen-free conditions
Male
0 - 999 days
140.8733
1.1851
129.0226
152.724
[mmHg]
ACI_0-79
ACI
ACI/NHok
ACI/NJcl
ACI/NKyo
ACI/NMna
ACI/NSlc
tail cuff photoplethysmography
specific pathogen-free conditions
Mixed
0 - 79 days
138.0636
1.0879
127.1842
148.9431
[mmHg]

E

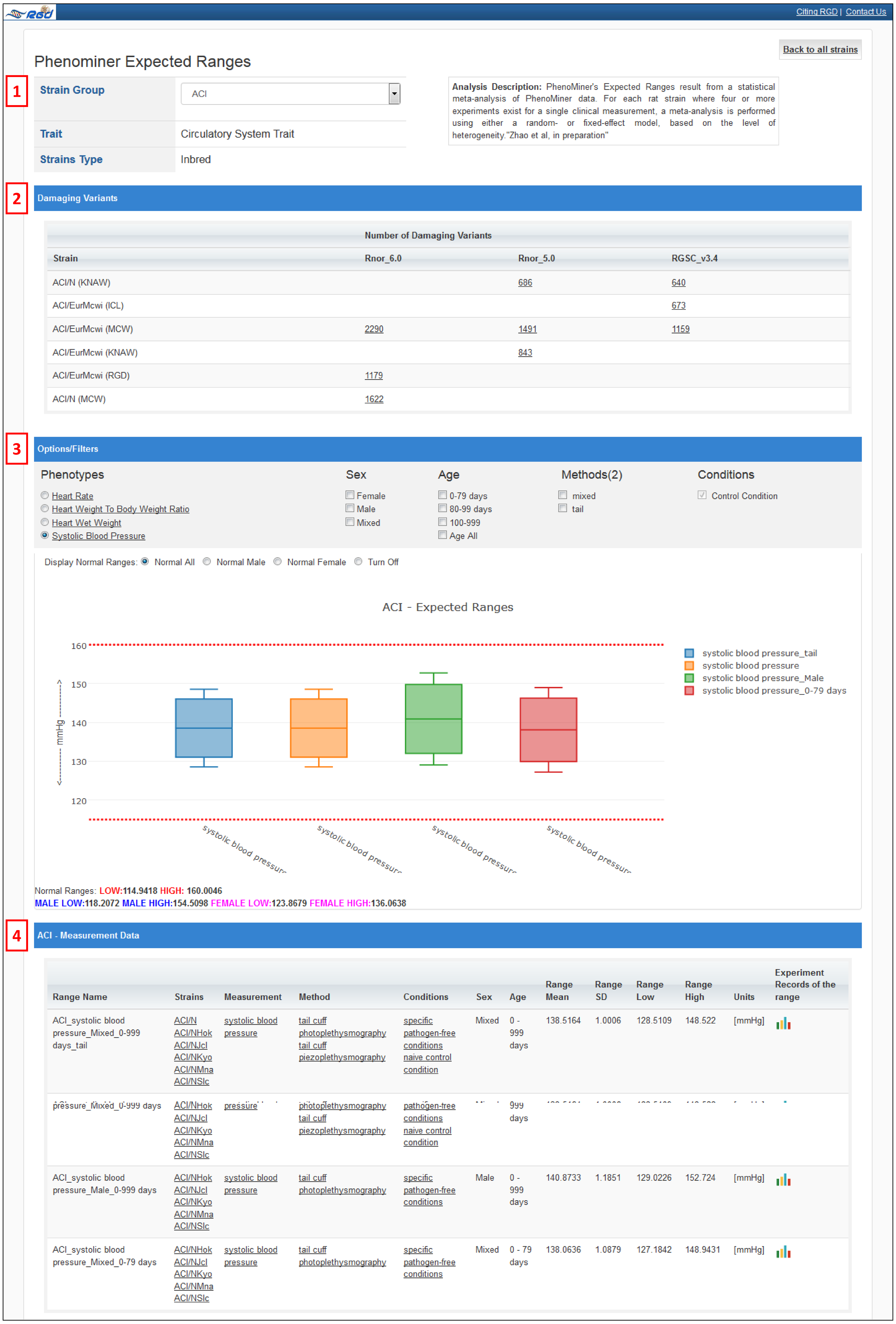

Supplementary Figure S5. The Expected Ranges tool. Expected ranges derived from a meta-analysis of RGD's PhenoMiner data are grouped using the Vertebrate Trait Ontology (A). Here "Circulatory system trait" has been selected (A1); expected ranges are also grouped and browsable by strain (A2) and (E). The top of the result page for the measurement "systolic blood pressure" shows the term, with a dropdown to select a different term in the category, and a panel with options for filtering the results by strain, age range, sex and method (B). Currently, only measurements taken under control conditions have been used for calculating the expected ranges, making the condition filter inactive. The graph of the expected ranges for systolic blood pressure shows the striking differences in blood pressure between normotensive strains like ACI and WKY and hypertensive strains such as SHR and SHRSP (C); "Normal Ranges", that is, the expected ranges for rat strains designated as normal controls by a domain expert, have been statistically determined for a number of measurements, including systolic blood pressure, and the normal range calculated using both male and female measurements is selected by default (C1); the normal range selected (here "Normal All") is shown as dotted lines in the graph, color-coded by the type of normal range displayed (C2); the values for the three normal ranges are shown below the graph (C3). The table below the graph shows the values of the expected ranges (D); for each strain group the specific substrains whose measurements were included in the calculations for that expected range are listed (D1); the "Studies within the range" column provides links to the PhenoMiner tool showing the individual values used in the calculation of that specific expected range. The "Strains" tab on the front page of the Expected Ranges tool links to a list of strains for which there are expected ranges. Here the strain group "ACI" has been selected (E). The top of the page lists the strain group, and trait from the original selection (E1); if any strain in the strain group has been sequenced, a list of the "samples", that is, the specific substrain that was sequenced and the group that performed the secondary analysis of the sequence, is displayed with counts of the total number of damaging variants for each assembly that link to lists of the variants and their positions (E2); as with the main expected ranges result page, the strain page shows a graph of the expected ranges for each applicable phenotype measurement (E3). Here systolic blood pressure has again been selected and the graph shows just the results for ACI; options for filtering the results are provided and the normal range for systolic blood pressure is shown; below the graph is a table of the expected range values for ACI and systolic blood pressure with details on the substrains, measurement method(s), conditions, sex and age, and a link to the PhenoMiner for the specific values included in the calculation (E4).

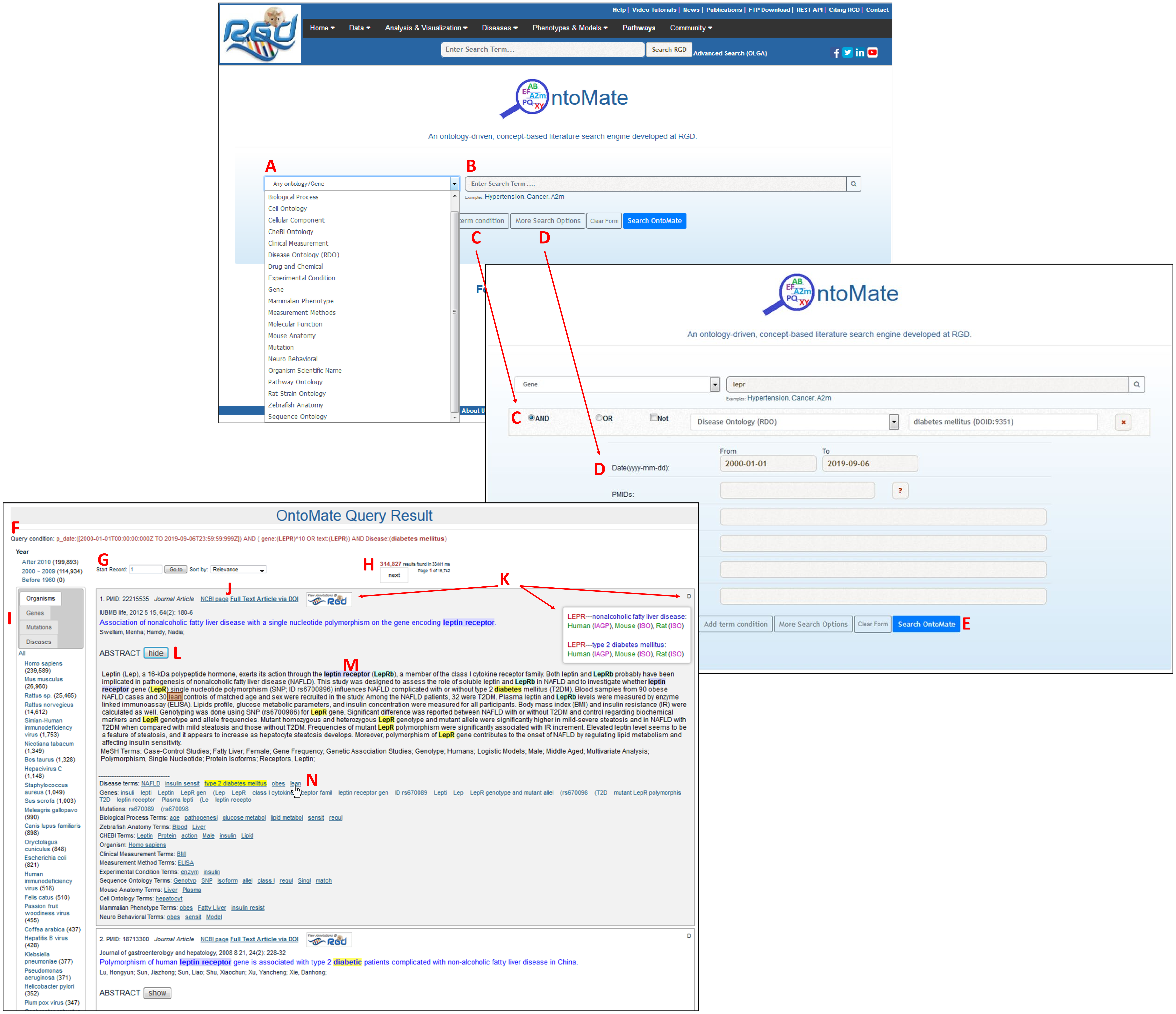

Supplementary Figure S6. The OntoMate text mining-based literature search tool. Ontology or gene specification (A); gene or term name – "Gene" and "Lepr" are selected in the second panel (B); "Add term condition" allows Boolean searches (AND, OR, NOT) for a second gene/selection, here Disease Ontology term "diabetes mellitus" is selected, so the search will be for Lepr and diabetes mellitus (C); additional search options can be added (D), here a date range has been entered; click "Search OntoMate" to execute the search (E); the top of the result page shows the specific Boolean search performed (F); options for navigating to a specific abstract and sorting (G); the count of the number of abstracts returned (H); filters with number of abstracts listed for each category, here the list gives options to limit the results to a specific organism from the NCBI taxonomy (I); links to PubMed abstract and full manuscript (J); annotation indicators showing that the reference is in RGD and has been used for disease annotations; mousing over the "D" shows the specific annotations from that publication (K); "Show/hide" button to display abstract (L); search terms and gene names are highlighted in the abstract by default (M); relevant terms are listed by category and linked to informational pages—search terms are highlighted in yellow, and mousing over a term in the list (for example, "lean") highlights that term in the abstract (N).

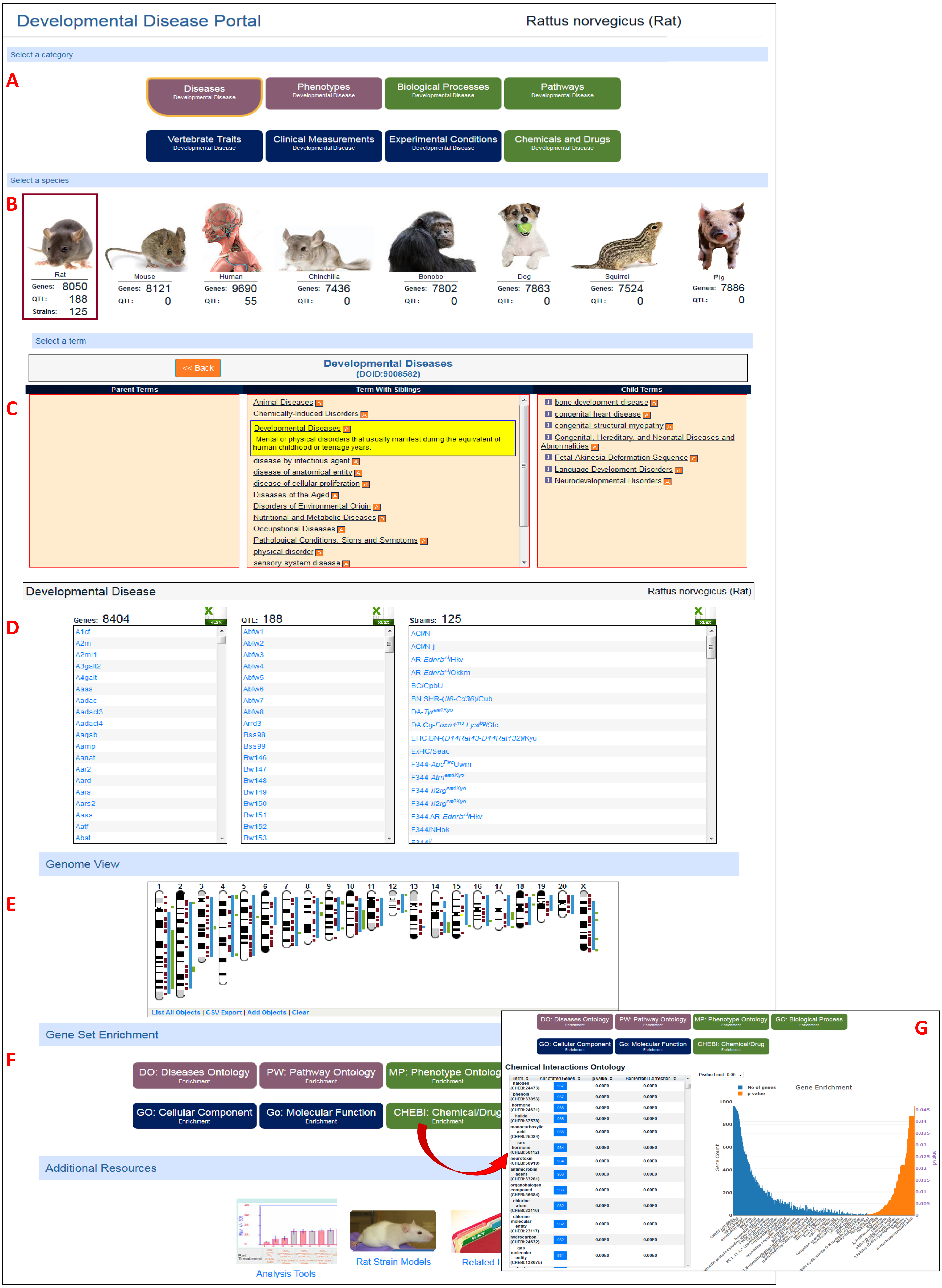

Supplementary Figure S7. The Developmental Disease Portal. The top section of the portal shows buttons to select an ontology category, here Diseases (A). Animal selection, here rat, with gene, QTL and strain numbers as applicable for each species (B). Ontology browser for the selected ontology (C). The ontologies have been trimmed to only include annotated terms, that is, terms where that term and/or one or more of its child terms have annotations. Downloadable lists of genes, QTLs and strains annotated to the selected term or category (D). Here the top-level term for the portal, "Developmental Diseases" has been chosen so the lists display all of the genes, QTLs and strains annotated to any developmental disease term. Genome Viewer display of objects annotated to the disease category (E). Genes are colored brown, QTLs are blue, and strains are green. Gene set enrichment ontology selection (F). Select an ontology to see the list of terms overrepresented in the annotations for genes in the list displayed above. Ontology enrichment display (G). The ChEBI ontology has been selected, opening an embedded version of the MOET tool which shows the enriched terms, their p-values and Bonferroni-corrected p-values, and a graph displaying the p-value and number of genes annotated to each term in the enrichment set.

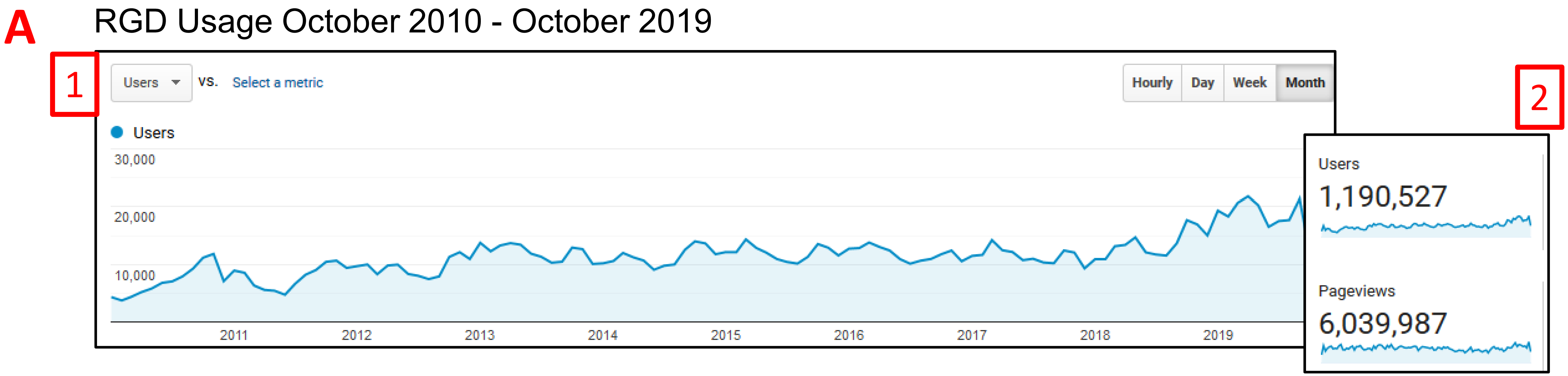

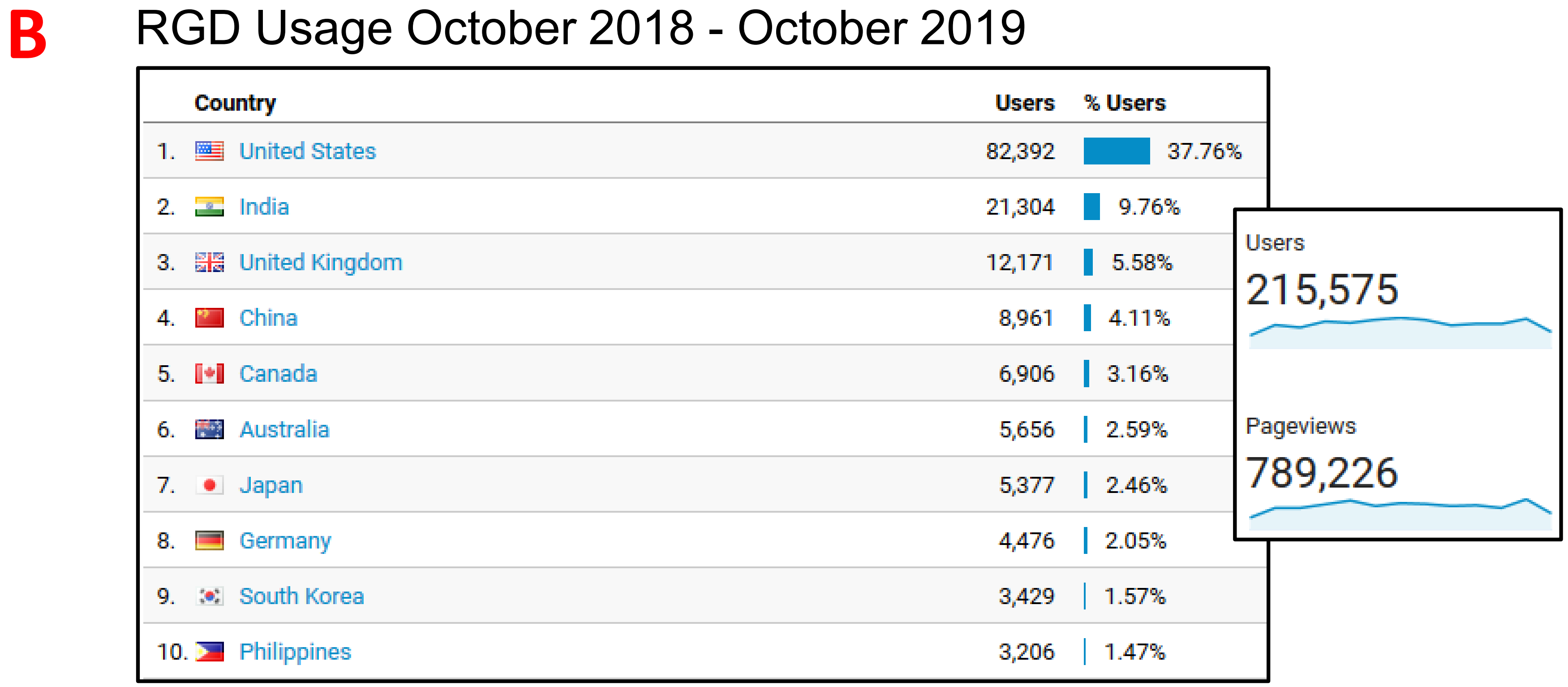

	Country	Users	% Users
1.	United States	82,392	37.76%
2.	India	21,304	9.76%
3.	United Kingdom	12,171	5.58%
4.	China	8,961	4.11%
5.	Canada	6,906	3.16%
6.	Australia	5,656	2.59%
7.	Japan	5,377	2.46%
8.	Germany	4,476	2.05%
9.	South Korea	3,429	1.57%
10.	Philippines	3,206	1.47%

Supplementary Figure S8. RGD Usage Statistics. Although usage statistics are not available for the entire 20 years of RGD's history, Google Analytics for the RGD website shows that the number of users has been increasing over the past 10 years (A1). Total number of users between 2010 and 2019 were almost 1.2 million and number of page views was over 6 million (A2). From October 2018 to October 2019, the total number of users was 215,575 and included individuals from the USA, Europe and Asia, while the total number of page views was 789,226 (B).
